# Non-stem cancer cell kinetics modulate solid tumor progression

**DOI:** 10.1186/1742-4682-8-48

**Published:** 2011-12-30

**Authors:** Charles I Morton, Lynn Hlatky, Philip Hahnfeldt, Heiko Enderling

**Affiliations:** 1 Center of Cancer Systems Biology, St. Elizabeth's Medical Center, Tufts University School of Medicine, 736 Cambridge Street, Boston, MA 02135, USA

**Keywords:** Cancer Stem Cells, Agent-Based Modeling, Solid Tumor Growth Kinetics

## Abstract

**Background:**

Solid tumors are heterogeneous in composition. Cancer stem cells (CSCs) are believed to drive tumor progression, but the relative frequencies of CSCs versus non-stem cancer cells span wide ranges even within tumors arising from the same tissue type. Tumor growth kinetics and composition can be studied through an agent-based cellular automaton model using minimal sets of biological assumptions and parameters. Herein we describe a pivotal role for the generational life span of non-stem cancer cells in modulating solid tumor progression *in silico*.

**Results:**

We demonstrate that although CSCs are necessary for progression, their expansion and consequently tumor growth kinetics are surprisingly modulated by the dynamics of the non-stem cancer cells. Simulations reveal that slight variations in non-stem cancer cell proliferative capacity can result in tumors with distinctly different growth kinetics. Longer generational life spans yield self-inhibited tumors, as the emerging population of non-stem cancer cells spatially impedes expansion of the CSC compartment. Conversely, shorter generational life spans yield persistence-limited tumors, with symmetric division frequency of CSCs determining tumor growth rate. We show that the CSC fraction of a tumor population can vary by multiple orders of magnitude as a function of the generational life span of the non-stem cancer cells.

**Conclusions:**

Our study suggests that variability in the growth rate and CSC content of solid tumors may be, in part, attributable to the proliferative capacity of the non-stem cancer cell population that arises during asymmetric division of CSCs. In our model, intermediate proliferative capacities give rise to the fastest-growing tumors, resulting in self-metastatic expansion driven by a balance between symmetric CSC division and expansion of the non-stem cancer population. Our results highlight the importance of non-stem cancer cell dynamics in the CSC hypothesis, and may offer a novel explanation for the large variations in CSC fractions reported *in vivo*.

## Background

The cancer stem cell hypothesis suggests that a subset of cells in a tumor is uniquely capable of driving disease. Definitively identifying cancer stem cells is often hailed as the Holy Grail of cancer research, as they are believed to be the sole initiator and driver of tumor growth, and thus their eradication may offer targeted tumor treatment. Characterization of cancer stem cells and quantification of their frequency within solid tumors, however, are topics still in their infancy and subjects of debate. Computational modeling in the context of the cancer stem cell hypothesis is an invaluable tool for assessing the relative contributions of basic cellular kinetics to macroscopic growth dynamics and composition of solid tumors. Two tumors, presenting the same cellular population size at clinical detection, can comprise very different subsets of cell types. As a result, the prognosis downstream of presentation could be very different. Unexpectedly, although cancer stem cells are the engine of tumor progression, the proliferative capacity of non-stem cancer cells appears to be modulating cancer stem cell kinetics and thus overall tumor progression dynamics. Understanding the oft-neglected contribution of the non-stem population within a heterogeneous tumor will help explain the large variations in cancer stem cell frequency reported in the literature, and ultimately help guide the design of appropriate treatments for patients with different disease presentations.

## Introduction

**Figure 1:**
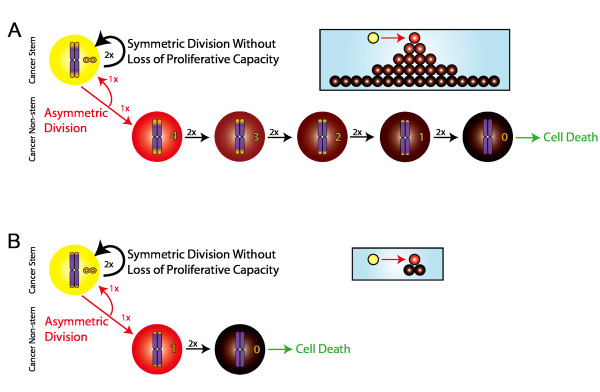
**Cancer stem cells (CSCs, yellow) may divide symmetrically (curved black arrows) to generate two identical CSCs; or asymmetrically (red arrows) to self-renew and yield a non-stem cancer cell with a discrete maximum proliferative capacity**. Non-stem cancer cells may divide only symmetrically (straight horizontal black arrows), with both parent and daughter experiencing a decrement in proliferative capacity. Mortal non-stem cancer cells with exhausted proliferative capacity die upon the ensuing division attempt. Non-stem cancer cells inherit generational life spans from the parent CSCs, resulting in a hierarchy depth (red to black gradient) that determines tumor population heterogeneity. Shown are hierarchical progressions of tumor populations originating from A) a CSC conferring ρ_max _= 4 and B) a CSC conferring ρ_max _= 1.

The cancer stem cell (CSC) hypothesis is a conceptual construct of tumor initiation, composition, and clinical response. Tumors are heterogeneous populations comprising clonogens (cells that can repeatedly initiate and maintain tumor clones) and cells devoid of clonogenic potential. The CSC hypothesis emerged following observation that the frequency of such clonogens can be remarkably low in solid tumors. Most ongoing discussions involve measurements of the frequency of CSCs, as broad ranges of CSC fractions often spanning multiple orders of magnitude have been observed in human solid tumors of various organ types [1]. CSCs are defined as immortal, possessing the ability to divide either symmetrically to yield two identical immortal cancer stem cells; or asymmetrically, to simultaneously self-renew and yield mortal non-stem cancer cells (CCs) with finite replicative potential. Each successive division of CCs reduces their remaining proliferative capacity by a finite amount, perhaps through a mechanism such as telomere shortening [2,3] (Figure 1). Eventually, progeny cancer cells without any remaining replicative potential arise and subsequent division attempts result in cell death.

The interplay of CSCs and CCs in tumor progression lends itself to quantitative agent-based modeling of tumor behavior using cellular automata, an established approach to analyze complex system dynamics and test systemic response to perturbation under a minimal set of rules and constraints [4-8]. Of particular intrigue is the paradoxical dependence of tumor progression on cell death within the CSC hypothesis, and the apparent disconnect between the clinical strategy of inciting cell death through chemical or radiological assault and the mathematical behavior of an untreated system that might well be self-limiting [9]. We have shown previously that a system looking solely at the balance between cell proliferation, migration, and death can be sufficient to explain anomalous features of the growth kinetics and morphology of simulated tumors [10]. In this model, without sufficient migration capability, a CSC may quickly become surrounded and spatially inhibited by non-stem CC progeny, and thus able to initiate only a clone of self-limiting size [11]. The resulting tumor achieves a pseudo-steady dormant state, characterized by a balance between cell death of CC with exhausted proliferative capacity at the periphery and subsequent proliferation of previously quiescent CC. Growth beyond the initial clone is impeded by the lack of space available for the CSC to divide potentially symmetrically to seed a new clone nearby - a mechanism others and we have described as self-metastatic tumor progression [10,12]. Herein we describe further exploration of cell-cell interactions underlying these phenomena toward describing tumor growth kinetics and CSC fraction as a function of the generational life span of the CCs. Depending on this inherited maximum proliferative capacity, CCs generate clonal population sizes that contribute to form smaller or larger tumors, respectively enabling or inhibiting CSC proliferation. Without sufficient telomerase activity, cells progressively shorten telomeres upon each division, eventually leading to terminal failure to replicate chromosomes and cell death [13]. The number of cell divisions is likely to be dependent on the function, morphology, age and developmental history of the specific organ. However, the specificity of telomere length to tissue type and the process of telomere shortening at a measurable rate [14] suggests that the remaining proliferative capacity of a given cell can be quantitatively modeled as the number of sequential mitoses until death (Figure 1). Agent-based models can capture the interactive consequences of cell-intrinsic properties and tumor population dynamics while allowing for the distinction of the participating CSC and CC compartments. Through stochastic simulations, we explore how altering CC generational life span influences tumor growth kinetics and CSC prevalence. CCs are thought to be the dominant population in a tumor, yet a large CC population impairs tumor progression *in silico *[9]. Therefore, non-linear modulation of tumor kinetics by the generational life span of CCs is expected. We identify optimal parameter values for tumor progression and inhibition, as predicted by the model, and discuss their biological applicability.

## Materials & methods

We extend an established agent-based cellular automaton model [9,10] of CSCs and CCs interacting in tumor growth. We assume that (i) CSCs are immortal with unlimited replicative potential, (ii) CC maximum proliferative capacity ρ_max _is inherited from the parent CSC following an asymmetric division, (iii) the probability of symmetric (p_s_) and asymmetric (1 - p_s_) CSC division is constant and stochastic, and (iv) cells require adjacent available space to migrate or proliferate. Individual cells are equipped with a cell cycle time of 24 hours and migration speeds of μ = 0, 50, 100, or 150 μm (i.e., 0, 5, 10, or 15 cell widths) per day [15]. We model migration speed as a trait inherent to a cell. While there are undoubtedly tissue types that naturally demonstrate faster or slower migration rates associated with the biological function of that organ system, or that change to a faster migration regime in a context such as wound healing, these tissue-level observations can be emergent properties of an agent-based model rather than imposed behaviors. How cell migration speed and environmental chemotactic gradients modulate tumor progression has been discussed elsewhere [10,16].

**Figure 2:**
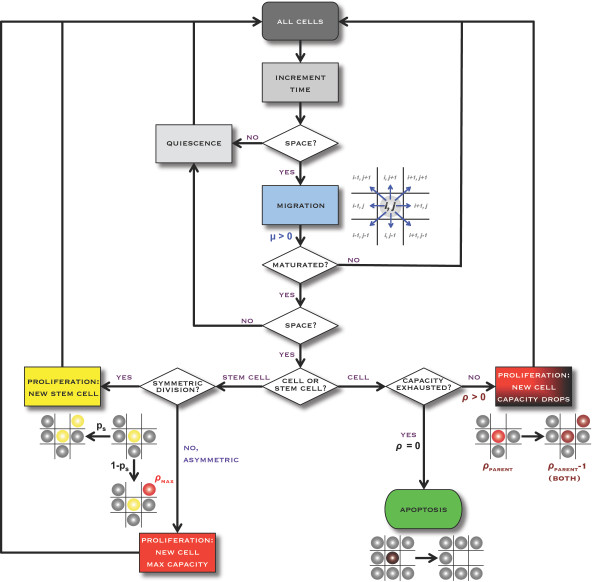
**Flow of control in the agent-based model**. All cells require available adjacent space to either migrate or proliferate, or they remain quiescent for the given time interval. Cells that have maturated through the cell cycle may proliferate, and the resulting progeny will initially occupy a neighboring grid locus. Non-stem cancer cells with no remaining replicative potential die upon a decision to proliferate, leaving the previously occupied grid locus empty.

We investigate proliferative capacities of CCs (that are first generation progeny of CSCs) of ρ_max _= {0-10}. To reflect the low frequency at which CSCs are observed [1], we set the probability of symmetric CSC division to p_s _= 1% or 10%. Symmetric CSC division yields two immortal CSCs. Asymmetric CSC division preserves the parent CSC and creates a CC with exactly proliferative capacity ρ_max_. While the CSC exhibits an immortal phenotype, through asymmetric division it endows the daughter CC with a discrete, finite replicative potential ostensibly proportional to remaining telomere length. This uniquely defines a maximum number of sequential mitoses this CC may undergo before exhausting its replicative potential. CSCs of different tissue origin and age may vary in telomere length and thus bequeath different maximum replicative potentials (ρ_max_) to their CC offspring. While fetal diploid cell strains in culture demonstrate a high number of divisions possible before mitotic arrest and culture degeneration (50 ± 10 population doublings, termed the "Hayflick Limit" [17,18]), 18 divisions have been reported for neutrophil granulocytes [19], and colonic crypt progenitors complete only four to six divisions [20]. Recent data indicates that that cancer cells have very short telomeres [21], and with telomere length being indicative of replicative potential, the generational life span of cancer cells might be shorter than widely assumed. CC division yields two CCs with decremented replicative potential ρ_new = _ρ_parent _- 1. Cells that have exhausted their proliferative capacity, i.e. ρ = 0, die upon the subsequent division attempt and are removed from the simulation. For each set of conditions, ten independent simulations were performed, each initialized by a single CSC at the center of a 350 × 350 computational lattice. Each lattice point represents a dimension of (10 μm)^2 ^that can be occupied by a single cell at most. The simulation time interval is one hour, with the probabilities of a proliferation or migration event during a given interval being equal to the cell cycle time and migration rate μ divided by 24 (hours per day). Tumor growth proceeds for five years of simulation time or until a total population size of 50,000 cells is reached. The stochastic behavior of the model is governed by the flowchart outlined in Figure 2.

Total number of cancer cells and number of cancer stem cells are recorded at simulated seven-day intervals. Simulation run times on a Quad-core 2.3 GHz Intel Xeon Mac OS and 64-bit CentOS servers range from ten minutes to seven days. Raw data from each simulation are analyzed to generate average growth curves and CSC fraction in the emerging tumors.

## Results

### Tumor growth kinetics display non-monotonic dependence on replicative potential

**Figure 3:**
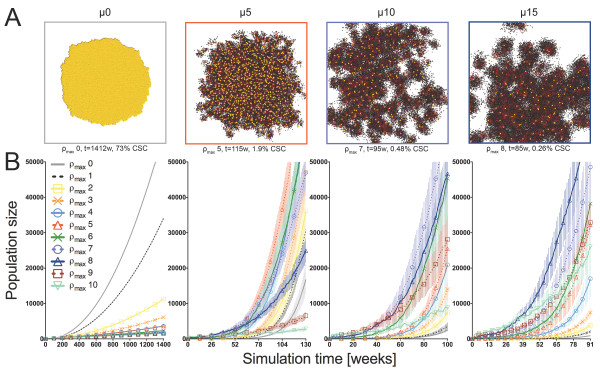
**Simulation results representing the set of conditions at which tumors most rapidly reach 5x104 total cell population when symmetric CSC division frequency ps = 1%.** A) Simulation results representing the set of conditions to most rapidly reach 5 × 10^4 ^total cell population at symmetric CSC division frequency p_s _= 1%. Shown from left to right: migration rates (μ) of 0, 5, 10, and 15 cell widths per day. Yellow CSCs enlarged for visibility. B) Corresponding growth curves for model tumors at all values of replicative potential ρ_max _= 0-10. Error bars correspond to SEM (n = 10).

If symmetric CSC division is a rare event (p_s _= 1%, Figure 3), tumors in which cells are unable to migrate (μ = 0) require more than 25 simulation years to reach 50,000 cells. In fact, migration has previously been identified as the pivotal mechanism to defeat intratumoral space constraints and thus allow for the symmetric CSC division events necessary for tumor expansion [10]. With low levels of cell migration, i.e. μ = 5 cell widths per day, tumors in which CCs have proliferative capacities of ρ_max _= 0-8 grow up to 50,000 cells before the five-year simulation threshold. Interestingly, tumors of cancer cells with longer generational life spans (ρ_max _= 9-10) fail to progress to that size within five years. Fastest tumor growth to 50,000 cells (115 ± 4 weeks [mean ± SEM]) is observed at intermediate CC proliferative capacity ρ_max _= 5. Over the same time interval, lower ρ_max _in CCs yields tumors of 6,008 cells (ρ_max _= 0) to 38,027 cells (ρ_max _= 4), and larger ρ_max _yields tumors of 37,829 to 2,310 cells (ρ_max _= 6 and ρ_max _= 10, respectively). We define the mean time required for the fastest growing member of each set of μ-p_s _pairs as t_critical _(in this case t_critical _= 115 ± 4 weeks), and summarize all results in Table 1.

**Table 1:** Summary of model tumor growth simulations across all conditions.

Symmetric division frequency	p_s _= 1%				p_s _= 10%			
**Migration rate (cell widths/day)**	**μ = 0**	**μ = 5**	**μ = 10**	**μ = 15**	**μ = 0**	**μ = 5**	**μ = 10**	**μ = 15**

Optimum ρ_max _(Figs. 3-4)	0	5	7	8	0	3	5	5

t_critical_ (weeks)	1303 ± 19	115 ± 4	95 ± 5	85 ± 6	130 ± 1	23 ± 1	17 ± 1	14 ± 1

CSC Fraction at optimum ρ_max_	73%	1.8%	0.5%	0.3%	76%	10%	3.1%	3.0%

ρ_max _pairs(Figs. 5-6)	n/a	2--8	3--10	5--10	n/a	2--5	3--7	1--9

ρ_max _pairs Sizes at t_critical_		1.7 × 10^4^	0.86 × 10^4^	2.1 × 10^4^		4.0 × 10^4^	3.8 × 10^4^	1.9 × 10^4^

CSC Fractions		13%--0.37%	6.5%--0.10%	1.6%--0.10%		17%--3.4%	9.5%--1.1%	30%--0.32%

Times to 5 × 10^4 ^cells (weeks)		140--172	124--196	102--128		25--25	18--19	18--21

Comparison of tumor growth parameters across symmetric division frequency probabilities (p_s_) and migration speeds (μ). For each value of migration rate and probability of symmetric CSC division a certain value for the proliferative capacity ρ_max _displayed the fastest expansion to the critical size of 5 × 10^4 ^cells. We defined the time at which that ρ_max _value reached that size threshold as t_critical _(simulation weeks, mean ± SEM, n = 10). Examination of the population sizes for the other ρ_max _values at t_critical _revealed that pairs of ρ_max _values on either side of the optimum had achieved similar population sizes by t_critical_, but in some cases would require distinctly different lengths of time to reach 5 × 10^4 ^cells.

Similar non-monotonic behavior is also observed at higher cell migration speeds (Figure 3; Table 1). At μ = 10, tumors reach 50,000 cells fastest if ρ_max _= 7 (t_critical _= 95 ± 5 weeks), with increasing tumor sizes from ρ_max _= 0 (1,977 cells) to ρ_max _= 6 (37,152 cells) and decreasing tumor sizes from ρ_max _= 8 (40,066 cells) to ρ_max _= 10 (7,840 cells). The higher migration rate leads to an increase in the CC generational life span that facilitates fastest tumor growth. A larger maximum proliferative capacity and the resulting increase in CC persistence are accommodated by faster cell migration that loosens the intratumoral spatial confinement. A further increase of cell migration speed to μ = 15 shifts the fastest growth to tumors with ρ_max _= 8 (t_critical _= 85 ± 6 weeks), again with consistently increasing tumor sizes as ρ_max _increases from 0-8 and decreasing tumor sizes as ρ_max _increases further.

**Figure 4:**
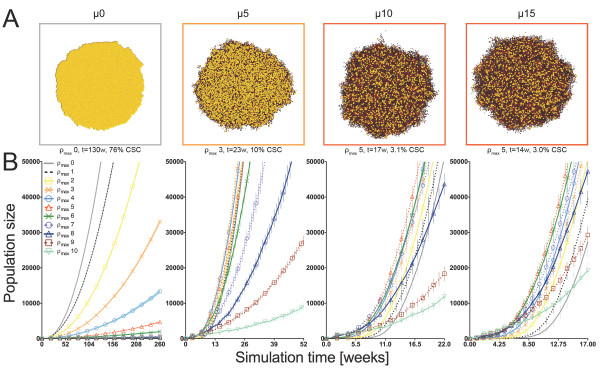
**Simulation results representing the set of conditions at which tumors most rapidly reach 5x104 total cell population when symmetric CSC division frequency ps = 10%.** A) Simulation results representing the set of conditions to most rapidly reach 5 × 10^4 ^total cell population at symmetric CSC division frequency p_s _= 10%. Shown from left to right: migration rates (μ) of 0, 5, 10, and 15 cell widths per day. Yellow CSCs enlarged for visibility. B) Corresponding growth curves for model tumors at all values of replicative potential ρ_max _= 0-10. Error bars correspond to SEM (n = 10).

Non-monotonic dependence on replicative potential is independent of the frequency of symmetric CSC division. With symmetric CSC division modeled at one order of magnitude higher frequency (p_s _= 10%, Figure 4), similar behavior is observed. Consistent with the immortality of CSCs driving macroscopic expansion, tumors grow faster. Interestingly, fastest tumor growth is observed with lower ρ_max _values compared to less frequent CSC division (p_s _= 1%, discussed above). Tumor growth peaks at ρ_max _= 3 for μ = 5, and ρ_max _= 5 for μ = 10 and μ = 15 (t_critical _= 23 ± 1, 17 ± 1 and 14 ± 1 weeks, respectively).

### Tumor CSC fraction correlates with growth kinetics downstream of a critical size

**Figure 5:**
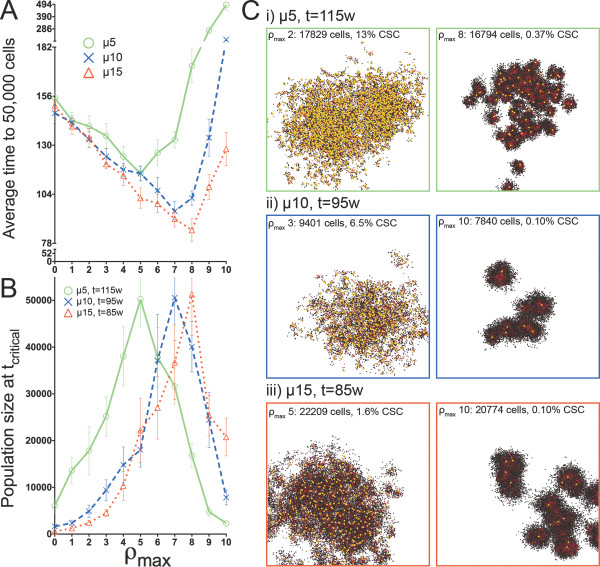
**Comparison of tumor populations across all generational life spans at tcritical when symmetric CSC division frequency ps=1%.** A) Time to simulation termination when p_s _= 1% and B) average population size at t_critical _for all ρ_max _values and migration speeds. Non-zero migration values demonstrate non-monotonic behavior and distinct optima at intermediate values of replicative potential. At t_critical_, model tumors from all ρ_max _values demonstrate a wide range of sizes. C) Matching pairs of model tumors for non-zero migration values differing only by the replicative potential of the seeding CSC. In most cases, morphologies are sufficiently distinguishable to enable estimation of CSC content and therefore the replicative potential of the cell of tumor origin. Top: μ = 5, left ρ_max _= 2, right ρ_max _= 8. Middle: μ = 10, left ρ_max _= 3, right ρ_max _= 10. Bottom: μ = 15, left ρ_max _= 5, right ρ_max _= 10. Yellow CSCs enlarged for visibility.

**Figure 6:**
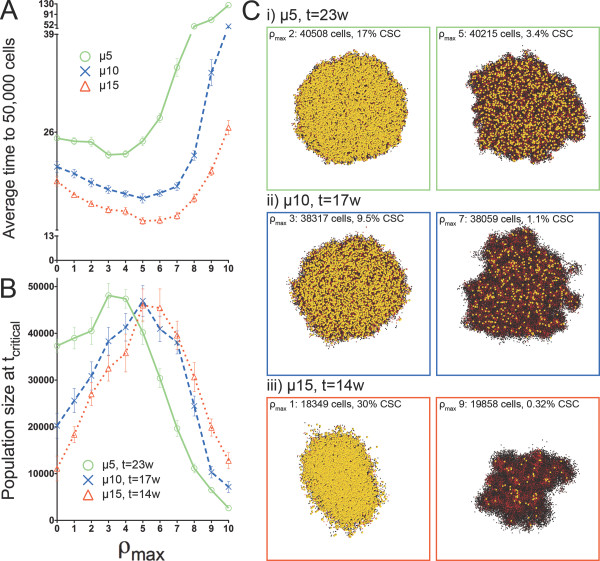
**Comparison of tumor populations across all generational life spans at tcritical when symmetric CSC division frequency ps=10%.** A) Time to simulation termination when p_s _= 10% and B) average population size at t_critical _for all ρ_max _values and migration speeds. Non-zero migration values demonstrate non-monotonic behavior and distinct optima at intermediate values of replicative potential. At t_critical_, model tumors from all ρ_max _values demonstrated a wide range of sizes. C) Matching pairs of model tumors for non-zero migration values differing only by the replicative potential of the seeding CSC. In most cases, macroscopic appearances are so similar that analysis for CSC content would be necessary to identify the replicative potential of the cell of tumor origin. Top: μ = 5, left ρ_max _= 2, right ρ_max _= 5. Middle: μ = 10, left ρ_max _= 3, right ρ_max _= 7. Bottom: μ = 15, left ρ_max _= 1, right ρ_max _= 9. Yellow CSCs enlarged for visibility.

Intuitively, higher frequencies of symmetric CSC division (p_s_) lead to larger CSC ratios. We have shown that intermediate CC generational life spans (ρ_max_) yield the fastest tumor growth (Figures 5 and 6), and lower or higher ρ_max _values slow tumor progression. Different CC maximum proliferative capacities on either side of the optimal ρ_max _for fastest tumor growth lead to tumors of comparable sizes but CSC fractions varying by in some cases multiple orders of magnitude (Table 1). Differing in morphology and CSC fraction, the growth trajectories of these tumors may cross, eventually going on to reach the 50,000-cell threshold in different lengths of time despite essentially identical net growth through the initial period.

When symmetric CSC division was comparatively rare (p_s _= 1%, Figure 5), tumors with very distinct morphologies and compositions were apparent on either side of the optimum ρ_max _for fastest growth. For example, at μ = 10, ρ_max _= 3 and ρ_max _= 10 tumors both reached ~0.86 × 10^4 ^cells in 95 weeks, but would require 124 or 196 weeks, respectively, to grow to 5 × 10^4 ^cells. With more frequent symmetric division (p_s _= 10%, Figure 6), morphologies were similar, but in some cases the differences in tumor composition and growth kinetics downstream of t_critical _were significant. For example, when μ = 15, tumors with CC that inherit either ρ_max _= 1 or ρ_max _= 9 could reach ~1.9 × 10^4 ^cells in 14 weeks, but would require 18 or 21 weeks, respectively, to then progress to 5 × 10^4 ^cells. In this case, morphologies at t_critical _were indistinguishable but the CSC fractions of 30% (ρ_max _= 1) and 0.32% (ρ_max _= 9) were dramatically different.

### Tumor CSC fractions are dependent on non-stem cancer cell replicative potential

**Figure 7:**
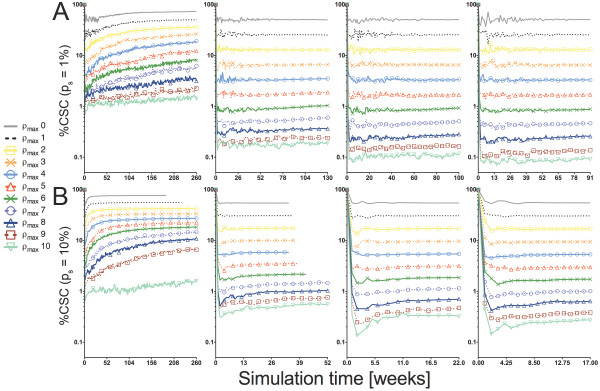
**The relative size of the CSC compartment of model tumors is inversely proportional to the generational life span of the non-stem cancer cell progeny**. Top row, p_s _= 1%; bottom row, p_s _= 10%. From left to right, μ = 0, 5, 10, and 15 cell widths/day. Model tumors seeded by CSC with low replicative potentials depended more strongly on symmetric division for macroscopic expansion and as such comprised a higher CSC fraction than those comprising deeper mortal non-stem cancer cell hierarchies. Early high amplitude fluctuations are attributable to the stochastic nature of growth, but in all cases, CSC fraction approaches a pseudo steady-state composition consistent with self-metastatic expansion.

The fraction of CSCs in tumors is directly dependent on the generational life span of CCs. With increasing ρ_max_, the CSC fraction decreases independently of any other parameter (Figure 7). When ρ_max _= 0, CCs die upon a first attempt at division, leading to a large CSC fraction. CCs, however, are still present as they arise from asymmetric CSC division with high frequency. Without migration, mature tumors comprised over 70% CSC at ρ_max _= 0. Increasing values of ρ_max _decreases CSC fractions to as low as 0.1% (ρ_max _= 10, μ = 15, and p_s _= 1%).

Cancer stem cell fraction fluctuates during early tumor growth due to the stochastic interpretation of (a)symmetric cancer stem cell division. Once a compositional pseudo equilibrium is established, tumor growth is dominated by a fractal self-similarity associated with self-metastatic progression as observed in previous modeling studies [10].

## Conclusions

The high variance in frequency of cancer cells expressing stem cell biomarkers within different tumors of the same tissue type can be attributed to intrinsic tumor hierarchy. While cancer stem cells (CSCs) are the engine of tumor progression [22], the contribution of non-stem cancer cells (CCs) to tumor growth kinetics is often disregarded, despite the fact that in many cases, these cells constitute the majority of the tumor. We set out to investigate if and how CCs modulate CSC dynamics and thus tumor progression. We utilized an agent-based model to describe the behavior of individual cells in response to their local environment, and for simplicity, focused only on the availability of space for cells to proliferate and migrate. Higher-order mechanisms, such as mechanical forces between cells and nutrient gradients, could be included in such a framework in the future to facilitate detailed understanding of how tumors overcome more specific, environmentally-imposed barriers to growth. Here we limited our analysis to the early stages of tumor development to sizes below angiogenic limitations [23] and focused on interaction of cells with only their nearest neighbors, which has been previously shown to be sufficient to reveal intriguing and often counterintuitive dynamics [9,10].

Much of the debate regarding the frequency of CSCs in various tumors derives from measurements of expression of cell surface proteins and/or gene expression profiling. While transformation at different locations on the somatic differentiation axis could lead to variable expression levels of stem cell markers following clonal expansion, our model suggests that the observed compositional heterogeneity could arise more simply from varying the generational life spans of the CC progeny, a trait inherited from asymmetric division of the CSCs driving tumor growth. As such, a transformation event yielding a CSC will confer a discrete maximum proliferative capacity to the resulting non-stem cancer cells based on a variety of factors, including tissue type [14] and host age [24-28]. In addition, heterogeneity of telomere lengths within the cells of a given organ at a certain age may lead to variability in tumor growth kinetics and composition following otherwise identical origin. We have shown a direct connection between the character of the CSC, the commensurate proliferative capacity of the non-stem cancer cells, and the macroscopic growth kinetics and compositional heterogeneity of the tumor.

Interestingly, simulated tumors arising from CSCs conferring intermediate maximum proliferative capacities demonstrated the most aggressive growth kinetics for non-zero migration rates, resulting in a non-monotonic dependence of the growth kinetics on the depth of the CC hierarchy. Macroscopically, this suggests an optimal balance between the freeing of space through death of cells with exhausted replicative potential and migration of CSCs to the resulting available space. The expansion of the immortal CSC compartment is critical for self-metastatic tumor progression [10]. In tumors with non-stem proliferative capacities either lower or higher than the optimum, macroscopic growth was less efficient. In the former case, the failure of CCs to persist before inevitable cell death slowed total population expansion, and space resulting from CC death opened up more quickly than the CSC population could exploit due to the infrequency of symmetric CSC division. In the latter case, enrichment of CSCs was inhibited by crowding of the surrounding CCs, whose long generational life spans inhibit cell activity in the tumor interior [5,9,11,29,30]. This bi-modal contribution of CCs to tumor growth and progression yields a non-monotonic dependence of tumor growth kinetics on CC generational life span. Optimum proliferative capacity is dependent on the interplay of other presumably independent tumor growth kinetic parameters, such as CSC symmetric division frequency and cell migration rate.

Tumors comprising CCs with generational life spans on either side of the optimum proliferative capacity can grow to similar sizes yet harbor CSC fractions that vary by multiple orders of magnitude, eventually resulting in vastly different growth kinetics. The degree to which two presenting tumors of identical size but distinct composition may respond differently to treatment must be considered. While tumors gaining size early through rapid expansion of CCs with longer generational life spans may contain only a small number of CSCs, CCs with shorter generational life spans yield later progressing tumors that may harbor many CSCs. These CSC-rich tumors have a significantly steeper growth curve at hypothetical time of detection and thus a worse prognosis. Comparatively self-limiting tumors arising from CSCs conferring high replicative potential to their mortal progeny should have a slower progression rate and thus experience an initial shrinkage in response to cytotoxic treatment, but the space opened up through killing of the mortal CCs might lead to enrichment of the immortal, self-metastatic CSC fraction at a rate faster than would occur naturally [31]. Conversely, tumors with already high fractions of CSCs are limited by the short life spans of the mortal progeny, such that a cytotoxic treatment would not exacerbate disease progression through selection for the immortal compartment.

The model presented herein is based on a small number of plausible, biologically motivated first-order assumptions. The interaction of cells has been restricted to their immediate neighborhood, and long-range interactions achieved through physical forces have been ignored for simplicity. Furthermore, we focused on initial tumor growth from a single cell to a small cluster that is thought to develop without nutrient deprivation or physical constraints enforced by the local host environment. These model assumptions apply to all simulations presented regardless of parameter values. As such, we believe is valuable to compare the time intervals for model tumor progression at different conditions in a qualitative sense only, as the translation from simulation times to biologically accurate growth kinetics would require more mechanistic detail.

Inclusion of extrinsic forces on tumor progression is expected to further enhance the observed behavior as modulators of the fundamental cellular level processes of proliferation, migration, quiescence, and cell death, all of which are already accounted for in the model. Further modeling efforts would be needed to explore the contribution of applied cytotoxic pressure (with varying degrees of differential susceptibility between the CSCs and mortal cancer cells) to overall growth kinetics and assess the role of stored information in the tumor microenvironment in determining decision pathway probability vectors at the cellular level.

While these model simulations begin with a single CSC, in a clinical setting, "time zero" for the patient and physician is the onset of disease presentation rather than the initiation event. Moreover, the number of human tumors that presents clinically is necessarily only the aggressive fraction of the total number of tumors existing, with a significant number likely being present in a dormant or slow-progressing state [32]. We speculate that solid tumors in a given organ with distinct CSC fractions may demonstrate substantially different progression patterns downstream of presentation and diagnosis, and that these differences may be attributable to the compositions in the respective populations as governed by CC generational life span. We believe that further exploration of the observed *in silico *correlation between CSC fraction and growth kinetics *in vivo *may help reveal tools that augment clinical predictive power.

## List of Abbreviations

CSC(s): cancer stem cell(s); CC(s): non-stem cancer cell(s); SEM: standard error of the mean.

## Competing interests

The authors declare that they have no competing interests.

## Authors' contributions

CM conceived of the study, executed the model simulations, analyzed the data, and drafted the manuscript. LH participated in the study coordination and helped draft the manuscript. PH participated in the study coordination and helped draft the manuscript. HE participated in the design and coordination of the study, executed the data visualization, and helped draft the manuscript. All authors read and approved the final manuscript.
